# Influence of the Surface Topography of Titanium Dental Implants on the Behavior of Human Amniotic Stem Cells

**DOI:** 10.3390/ijms25137416

**Published:** 2024-07-06

**Authors:** Rodrigo Riedel, Soledad Pérez-Amodio, Laura Cabo-Zabala, Eugenio Velasco-Ortega, Julieta Maymó, Javier Gil, Loreto Monsalve-Guil, Iván Ortiz-Garcia, Antonio Pérez-Pérez, Victor Sánchez-Margalet, Alvaro Jiménez-Guerra

**Affiliations:** 1 Departament Química Biológica, Facultad de Ciencias Exactas y Naturales, Universidad de Buenos Aires, Ciudad Universitaria Pabellón 2, 4° Piso, Buenos Aires 1428, Argentina; riedelrodrigo@gmail.com (R.R.); jmaymo@qb.fcen.uba.ar (J.M.); 2 CONICET, Instituto de Química Biológica de la Facultad de Ciencias Exactas y Naturales (IQUIBICEN), Universidad de Buenos Aires, Ciudad Universitaria Pabellón 2, 4th Floor, Buenos Aires 1428, Argentina; 3 Bioengineering Institute of Technology, Facultad de Medicina y Ciencias de la Salud, Universidad Internacional de Cataluña, 08195 Sant Cugat del Vallés, Spain; sperezam@uic.es; 4 Sección de Inmunología, Hospital Regional Universitario de Malaga, Instituto de Investigacion Biomédica de Malaga (IBIMA), 29590 Málaga, Spain; arual_cz@hotmail.com; 5 Department of Stomatology, Faculty of Dentistry, University of Seville, 41004 Sevilla, Spain; evelasco@us.es (E.V.-O.); ivanortizgarcia1000@hotmail.com (I.O.-G.); alopajanosas@hotmail.com (A.J.-G.); 6 Departamento de Bioquímica Médica y Biología Molecular e Inmunología, Hospital Universitario Virgen Macarena, Facultad de Medicina, Universidad de Sevilla, Avenida Sánchez Pizjuán 4, 41009 Sevilla, Spain; aperez14@us.es (A.P.-P.); margalet@us.es (V.S.-M.)

**Keywords:** titanium, surfaces, amniotic stem cells, dental implants

## Abstract

The dental implant surface plays a crucial role in osseointegration. The topography and physicochemical properties will affect the cellular functions. In this research, four distinct titanium surfaces have been studied: machined acting (MACH), acid etched (AE), grit blasting (GBLAST), and a combination of grit blasting and subsequent acid etching (GBLAST + AE). Human amniotic mesenchymal (hAMSCs) and epithelial stem cells (hAECs) isolated from the amniotic membrane have attractive stem-cell properties. They were cultured on titanium surfaces to analyze their impact on biological behavior. The surface roughness, microhardness, wettability, and surface energy were analyzed using interferometric microscopy, Vickers indentation, and drop-sessile techniques. The GBLAST and GBLAST + AE surfaces showed higher roughness, reduced hydrophilicity, and lower surface energy with significant differences. Increased microhardness values for GBLAST and GBLAST + AE implants were attributed to surface compression. Cell viability was higher for hAMSCs, particularly on GBLAST and GBLAST + AE surfaces. Alkaline phosphatase activity enhanced in hAMSCs cultured on GBLAST and GBLAST + AE surfaces, while hAECs showed no mineralization signals. Osteogenic gene expression was upregulated in hAMSCs on GBLAST surfaces. Moreover, α2 and β1 integrin expression enhanced in hAMSCs, suggesting a surface−integrin interaction. Consequently, hAMSCs would tend toward osteoblastic differentiation on grit-blasted surfaces conducive to osseointegration, a phenomenon not observed in hAECs.

## 1. Introduction

Osseointegration is the key to good fixation and long-term performance of dental implants. According to Albretksson et al., there are six factors that affect osseointegration, including the implant manufacturing material, the surface, the mechanical properties of the bone, the dental implant design, the masticatory loads, and the surgical technique [1,2,3,4]. Many studies have shown the significant importance of the implant surface, where a suitable topography for osteoblastic adhesion, proliferation and differentiation has been established [5]. However, there are other properties such as wettability and surface energy with its polar and dispersive components that will influence metabolic activity [6,7,8].

The different surfaces obtained by sandblasting, acid-etching, and those treated by sandblasting and subsequent acid-etching have been deeply studied in the aspects of physicochemical properties of the surface [9,10,11]. Today, the most common surfaces in implant dentistry are those treated by the projection of abrasive particles on the titanium (Ti) surface, especially alumina particles. This treatment makes it possible to obtain the desired roughness for the cells since it can be adjusted by the size of the abrasive particles, projection pressure, distance from the gun to the surface and the abrasiveness of the particles. In addition to achieving the desired roughness, the sandblasting treatment produces a compressive residual stress on the surface of the dental implant that hinders the nucleation of cracks due to fatigue, resulting in better long-term mechanical behavior. Different treatments will produce different wettability properties and surface energies that will also affect cell behavior [12,13,14,15,16]. This blasted surface can be modified with an acid-etching procedure. Strong acids like hydrochloric, sulfuric, and nitric acids are normally used for etching. The topography of both sandblasted and acid surfaces showed irregularities such as large dips, small micropits, sharp edges, and pointed tips. Sa, one of the surface parameters defined as the arithmetic mean height of the surface, is approximately 1.5 µm to 2 µm. It is known that osteogenic cells migrate to a roughened Ti surface through the fibrin clot and seem to recognize the irregularities of the surface [17,18,19].

Performing topography and surface chemistry modifications on titanium implants enhances osseointegration by increasing surface tension. In this context, grit blasting has been shown to improve the biological activity of titanium surfaces through increased microhardness, which is linked to higher compressive surface tension, greater hydrophilicity, and enhanced osteoblast activity. Additionally, compressive stress from grit blasting enhances implant fatigue life by inhibiting crack formation [20,21]. However, there are risk factors for biological and mechanical complications, contributing to late implant failure [22,23,24,25,26,27].

Recent studies have reported that the osteogenic effect of stem cells around an implant was clearly identified, showing the greater new bone formation especially in the early phase of bone regeneration. Moreover, stem cells can develop a direct conversion to osteoblasts, and in the early stages of osseointegration, stem cells can stimulate bone formation through a direct role in the source of new osteoblasts. This osteogenic capacity of stem cells can be improved in relation to implant surface type by an additional effect of sandblasting and acid-etching surfaces at a later phase of bone healing [28,29,30].

The amnion of the human placenta is a source of multipotent and pluripotent stem cells. The unlimited potential to provide cells, easy accessibility, minimal or no ethical and legal issues associated with its use, and cell isolation without invasive procedures makes the amnion very attractive for regenerative medicine applications [31]. Placental stem cells such as human amniotic mesenchymal stem cells (hAMSCs) and human amniotic epithelial stem cells (hAECs) can be isolated from the human amniotic membrane. Amnion cells possess immunomodulatory and anti-inflammatory properties [32], as well as differentiation potential, including osteogenic lineage [33,34,35,36]. Moreover, they express embryonic and pluripotency markers, do not express telomerase, and are non-tumorigenic [37]. These properties make the amniotic stem cells a valuable and non-controversial tool for application in tissue engineering and dental implanting.

The aim of this study was to compare the response of Human Amniotic Epithelial and Mesenchymal Stem Cells on four different Ti surfaces: (i) machined acting (MACH), (ii) grit-blasted (GBLAST), (iii) acid etching (AE) and (iv) grit-blasted and acid etching (GBLAST + AE) through the evaluation of cell viability, ALP activity, calcium deposition, and osteogenic marker expression. Understanding the cellular behavior of amnion stem cells on the Ti surface will provide new knowledge to facilitate the development of stem cell-based therapy applied in oral implantation.

## 2. Results

### 2.1. Topography of the Samples

**Figure 1:**
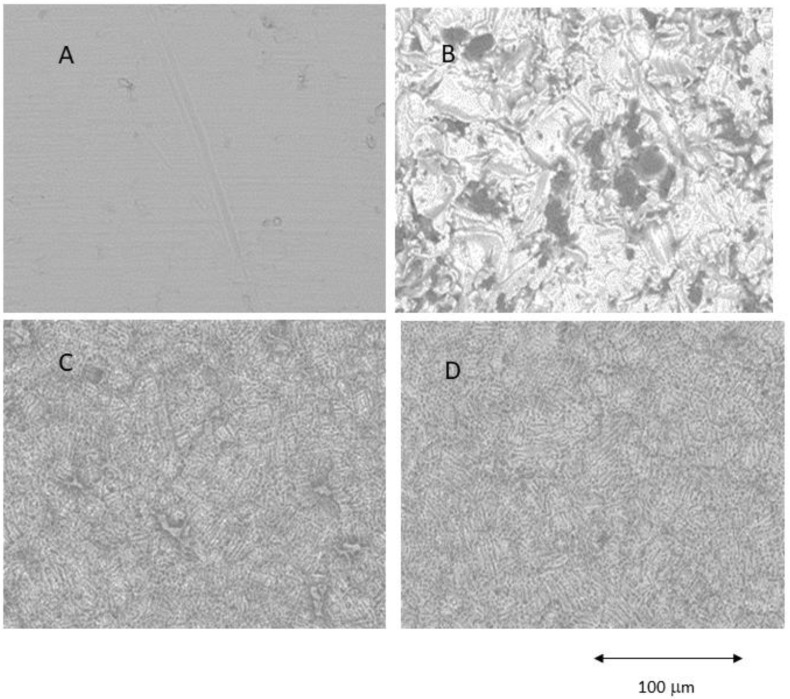
Different surfaces observed by Scanning Electron Microscope. (**A**) Machined (MACH), (**B**) Grit Blasting (GBLAST), (**C**) Grit Blasting and Acid Etching (GBLAST + AE), (**D**) Acid Etched (AE).

**Figure 2:**
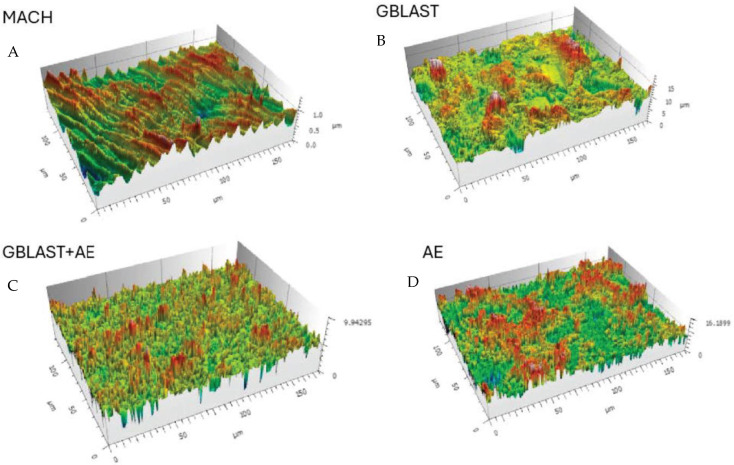
Surfaces of the different treatments observed by Confocal Laser Scanning Microscopy images. (**A**) Machined (MACH), (**B**) Grit Blasting (GBLAST), (**C**) Grit Blasting and Acid Etching (GBLAST + AE), (**D**) Acid Etched (AE).

Figure 1 shows Confocal Laser Scanning Microscopy images of the different roughnesses obtained. The main surface properties (i.e., roughness, contact angle) of the study groups are summarized in Table 1 and the profiles obtained by interferometric microscope in Figure 2.

In Table 2 can be observed the values of the free surface energy with the two components: polar and dispersive.

The results showed that the acid-etched surfaces produce a higher roughness than machined samples. The discs treated with GBLAST show higher roughness than AE and MACH. GBLAST surfaces presented sharper valleys and peaks and some residues of the aluminum oxide used for abrasive particle projection. GBLAST followed by AE showed that the GBLAST roughness is maintained, but the sharp angles of the roughness are reduced due to the etching acid. A microroughness produced by AE is observed in the macroroughness obtained by sandblasting.

X-ray energy dispersive microanalysis studies for all GBLAST and GBLAST + AE samples indicate that the amount of alumina is lower than 4% on the titanium surfaces. No other contamination was detected on any of the other surfaces.

The microhardness results indicate values for MACH surfaces of 178 HVN (sd 23), for AE surfaces of 188 HVN (sd 17), for abrasive spray-treated GTBLAST of 265 (sd 23), and for GBLAST + AE of 268 (sd 23). These results indicate significant differences between the MACH and AE surfaces with respect to the GBLAST and GBLAST + AE. This can be explained by the co-pressure exerted on the projection of the abrasive particles. Between MACH and AE, as well as between GBLAST and GBLAST + AE, the differences were not statistically significant (*p* < 0.001).

### 2.2. Biological Characterization of Titanium Disc Surfaces

#### 2.2.1. Cell Viability

**Figure 3:**
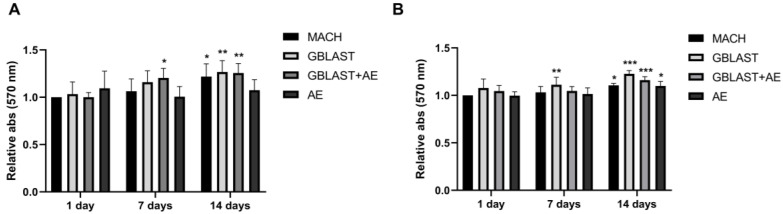
Cell viability of (**A**) hAMSCs and (**B**) hAECs cultured on different titanium surfaces. Data are presented as mean ± SD. * *p* < 0.05, ** *p* < 0.01, *** *p* < 0.001 vs. control day 1.

HAMSCs and hAECs were cultured on titanium surfaces for 1, 7, and 14 days. A significant increase in cell viability was observed in hAMSCs on GBLAST surfaces after culturing for 7 days, measured by MTT assay. After 14 days of culture, cell viability increased in MACH, GBLAST, and GBLAST + AE surfaces compared with the control of day 1 (Figure 3A). Similar results were observed when hAECs were cultured on the different Ti disc surfaces (Figure 3B).

#### 2.2.2. Alkaline Phosphatase Activity

**Figure 4:**
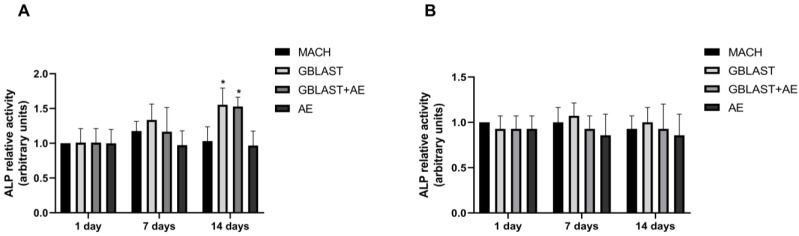
ALP activity in (**A**) hAMSCs and (**B**) hAECs cultured on different surfaces for 1, 7 and 14 days. Data are presented as mean ± SD. * *p* < 0.05 vs. control day 1.

ALP activity serves as a marker for monitoring the early stages of osteoblasts. The enzyme activity was measured to investigate the osteogenic differentiation of human amnion stem cells cultured on the different Ti disc surfaces. At day 14, ALP activity was significantly enhanced in hAMSCs cultured on GBLAST and GBLAST + AE-treated surfaces compared to the MACH disc of day 1 (Figure 4A). On the contrary, when evaluating ALP activity in hAECs culture, there were no significant differences on any of the surfaces studied (Figure 4B).

#### 2.2.3. Calcium Intake

**Figure 5:**
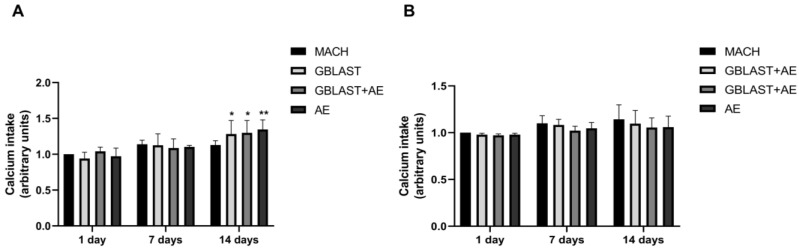
Calcium intake from (**A**) hAMSCs and (**B**) hAECs cultured on different surfaces for 1, 7 and 14 days. Data are presented as mean ± SD. * *p* < 0.05, ** *p* < 0.01 vs. control day 1.

The calcium intake, indicative of the late-stage osteogenic differentiation expressed by mature osteoblasts during bone formation, was determined in culture media from hAMSCs and hAECs at 1, 7, and 14 days. As shown in Figure 5A, the calcium intake from hAMSCs was significantly increased in GBLAST, GBLAST + AE, and AE-treated surfaces at 14 days of culture. No statistically significant differences in calcium levels were found in hAEC cultures (Figure 5B).

#### 2.2.4. Osteogenic Gene Expression

**Figure 6:**
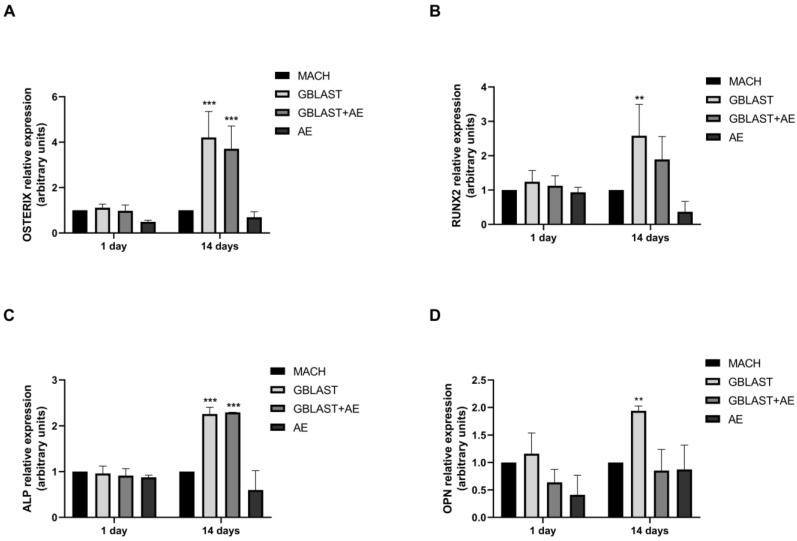
Quantitative RT-PCR results of mRNA osteogenic gene expression in hAMSCs, (**A**) OSTERIX, (**B**), RUNX2, (**C**) ALP and (**D**) OPN. Data are presented as mean ± SD. ** *p* < 0.01, *** *p* < 0.001 vs. respective control.

Expression of osteogenic markers, such as OSTERIX, runt-related transcription factor 2 (RUNX2), alkaline phosphatase (ALP), and osteopontin (OPN), was evaluated by qRT-PCR in hAMSCs at 1 and 14 days of culture. In Figure 6, there is a significant increase in the expression levels of OSTERIX, RUNX2, ALP, and OPN in hAMSCs that were cultured on GBLAST surfaces for 14 days. In addition, the combination of both GBLAST and AE treatments induces a significant increase in OSTERIX and ALP expression (Figure 6A,C).

#### 2.2.5. α and β Integrin Subunit Gene Expression

**Figure 7:**
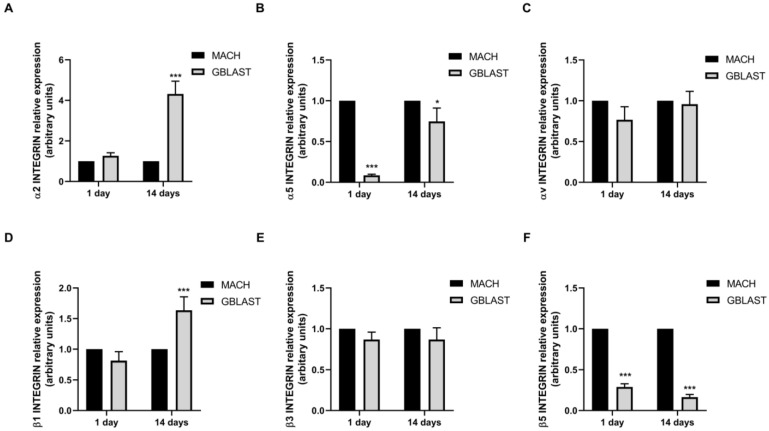
Quantitative RT-PCR results of mRNA integrin α and β subunit gene expression in hAMCs, (**A**) α1, (**B**) α5, (**C**) αv, (**D**) β2, (**E**) β3, and (**F**) β5. Data are presented as mean ± SD. * *p* < 0.05, *** *p* < 0.001 vs. respective control.

Integrins play a crucial role in the attachment of cells to proteins that are adsorbed on the surface of an implant (Figure 7). To further investigate the interaction mechanisms between hAMSCs and GBLAST surface, we analyzed the gene expression of α and β integrin subunits, measured by qRT-PCR. Figure 7A,D showed that only α2 and β1 integrin subunits were upregulated in hAMSCs at 14 days on the GBLAST surface, compared to the machined acting surface.

## 3. Discussion

The present study reports the impact of four different titanium surfaces on the biological behavior of human amniotic membrane-derived stem cells. Our findings indicate that Ti surface implants with different surface treatments affect the proliferation and differentiation of hAMSCs and hAECs. These treatments of titanium surfaces, which bring about modifications of topography, hydrophilicity, and chemical properties, positively affect hAMSCs and hAECs responses. We found that GBLAST surfaces could enhance osteoblast differentiation of hAMSCs, benefiting osseointegration.

The surface topography and physiochemical properties of Ti implants are crucial characteristics for osseointegration. The microscopic topography features can increase osseointegration by direct bone-to-implant contact and resistance to functional loads on titanium surfaces. Moreover, modifications to the surface, affecting both topography and surface chemistry, have been reported to be an important influence regarding adhesion, proliferation, and differentiation of specific cells (i.e., osteoblasts) via a direct interaction between cells and the implant surface [5,9,13]. In fact, several surface treatments (i.e., sandblasting, acid-etching,) have been developed to improve a better and faster bone formation in experimental and clinical studies [14,17,38].

The results of this study demonstrated by the topography are observed via the use of a Scanning Electron Microscope and confirmed by Confocal Scanning Microscopy. The different profiles show that the highest roughness surface was obtained by the grit blasting treatment; however, the analysis showed that acid etching slightly reduces the surface roughness. According to several reports, the roughness levels obtained by GBLAST and GBLAST + AE treatments are in the range of optimal roughness for osteoblast cell adhesion [17,19,25]. Our results show higher cell viability for GBLAST and GBLAST + AE surfaces compared to machine-made and acid-etched surfaces. This can be explained by the fact that surfaces with high surface energy promote cell adhesion. In this regard, it has been observed that osteoblasts cultured in rough surfaces such as GBLAST + AE interact with this type of surface through filopodia [39,40].

This study also shows that roughness produces higher contact angle values on surfaces treated by grit blasting, indicating higher hydrophobicity.

The values of the rough surfaces have been angle-corrected according to the Wenzel equation [41,42,43]. The surface energy can be an aspect that will favor osteoblastic cell activity. Surface energy/wettability indicates the balance between the intermolecular interactions when a solid surface and a liquid are brought into contact. Higher surface energy/wettability is known to promote the differentiation of mesenchymal stem cells to an osteogenic lineage as well as facilitate implant-to-bone contact. The modifications of surface morphology by roughening can change the surface charge and energy and, in this way, promote the biological response of cells. Several reports indicate that implant surfaces having higher surface energy/wettability are more suitable for cell adhesion and proliferation compared to surfaces presenting lower surface energy [44,45].

The microhardness levels are also higher on surfaces treated with grit-blasting because the projection of abrasive particles causes a plastic deformation of the titanium generating a state of residual compressive stress. This residual stress improves the mechanical properties of fatigue as it inhibits the formation of cracks on the metal surface [13,39].

The surface properties of titanium implants such as topography, roughness, wettability, and chemistry have a significant impact on cell activity [5,6,9]. Wang et al. [46] demonstrated that the gradient nanostructured Ti surfaces promoted the adhesion and proliferation of hAMSCs in vitro. Moreover, the morphology of hAMSC on the surfaces exhibited robust spreading and extended pseudopods. Our study reported that hAMSCs and hAECs were successfully cultured on titanium surfaces for 1, 7 and 14 days. A significant increase in cell viability was observed in hAMSCs on GBLAST surfaces after culturing for 7 days. After 14 days of culture, cells viability increased in MACH, GBLAST and GBLAST + AE treated surfaces. Similar results were observed when hAECs were cultured on different surfaces.

This study analyzed the effects of Ti-surface implants with different surface treatments on the proliferation and differentiation of hAMSCs and hAECs. Our in vitro results confirm that the treatment of the titanium surface, which brings about modifications to the topography, hydrophilicity, and chemical properties of the titanium implants, is positive for hAMSCs and hAECs responses. These biological findings are noteworthy as we have demonstrated the robust cell viability of amniotic stem cells over all analyzed surfaces.

In fact, numerous in vitro studies confirmed the effect of the modifications of topography and physiochemical properties of titanium surfaces on stem cell behavior and osteogenesis [28,29,30,46]. Several surfaces (i.e., sandblasting, acid-etching, zirconia) have demonstrated improved osseointegration through the direct stimulation of the microenvironment of stem cells. It has been recently reported that titanium implants with different surface roughness, nanostructures, and wettability, fabricated by the modification of sandblasted and acid-etched titanium by H_2_O_2_ treatment, significantly enhance cell adhesion spreading and proliferation [28]. Furthermore, early cell adhesion, migration, and efficient differentiation of stem cells to osteoblasts are key to colonizing the surface of dental implants. In addition, all the above-mentioned cell events prevent biofilm formation and implant-associated infection. Moreover, micro–nano-roughness of titanium results in faster maturation of osteoblasts/osteocytes and osteogenic differentiation of Mesenchymal Stem Cells (MSCs) [29].

We evaluated mRNA expression levels of certain master regulator genes of osteoblast differentiation (RUNX2 and OSTERIX) in hAMSCs at 14 days of culture. RUNX2 and OSTERIX are transcription factor genes for osteogenic differentiation. It has been demonstrated that RUNX2 is essential for the MSC differentiation into preosteoblasts, and OSTERIX is an osteoblast-specific transcription factor for the differentiation of preosteoblasts into mature osteoblasts [45]. In this context, our study shows that GBLAST and GBLAST + AE surfaces produced an increase in RUNX2 and OSTERIX expression by 4,2-fold and 3,7-fold, respectively, compared to the control surface. These findings indicate that a surface with GBLAST modification is effective in initiating the first step in the osteogenesis-related cascade of hAMSCs, promoting the upregulation of critical bone-specific transcription factors. We also quantified the mRNA expression of some phenotype marker genes that indicate the stages of early (ALP) and mature (OPN) osteoblast differentiation. After 14 days of incubation, we observed an increased expression of ALP in GBLAST and GBLAST + AE surfaces, indicating early osteoblastic differentiation on both treated surfaces. However, it appears that the late stages of osteoblastic differentiation, detected by OPN expression, only occur on GBLAST surfaces at 14 days of culture. This result suggests a potential delay in the progression of osteoblast differentiation on surfaces treated with GBLAST + AE, possibly requiring a prolonged culture period for completion. None of the evaluated osteoblast genes were detected in hAECs during the 14 days of culture.

Alkaline phosphatase (ALP) is a commonly used marker of osteoblast differentiation. This enzyme plays a role in extracellular matrix formation, maturation, and mineralization by enhancing the concentration of phosphate ions and inhibiting phosphoric ester action [47,48]. Our results show that after 14 days of hAMSCs culture, ALP activity significantly increased on GBLAST- and GBLAST + AE-treated surfaces. The absence of ALP enzyme activity in the supernatant of hAEC cultures indicates that these cells did not differentiate into osteoblasts on any of the treated surfaces used in our study. The fact that only hAMSCs showed osteogenic differentiation when cultured on the treated surfaces can be explained by the differences in osteogenic differentiation capacity already reported between these cells and hAECs. Si et al. [49] found that although hAECs displayed osteoblastic differentiation capacity, hAMSCs showed a higher osteoblastic phenotype when cultured under osteogenic stimulation. These authors reported lower ALP activity and extracellular mineralization of hAECs compared to hAMSCs. Based on this previous evidence, it is possible that hAECs do not respond to the different surfaces by changing their phenotype to a more osteoblastic one as observed by hAMSCs.

Mineralization activity is a fundamental late-phase sign of osteogenic differentiation [50]. Yin et al. [51] showed that hAMSCs accelerated mineralized deposition rates on implant surfaces injected into rabbit maxillary sinus floor elevation models. Moreover, hAMSCs improved bone formation differentiating into osteoblast cells. As we expected, calcium intake from hAMSCs was significantly increased at day 14 in GBLAST, GBLAST + AE, and AE-treated surfaces. In line with the previous results on ALP activity, calcium levels were not detected in hAEC cultures.

It is known that surface roughness influences the concentration and biological activity of adsorptive proteins and subsequent cell behavior [52]. Integrins are the links between cells and the extracellular matrix and play a crucial role in signal transducers for regulating cell growth, differentiation, and motility [53]. Olivares-Navarrete et al. [54] demonstrated that α2β1 integrin, which binds to sites on type I collagen, mediates the osteoblast response to micro and nanostructured Ti implant surfaces in MSCs. Similarly, the introduction of titanium surface microroughness decreased the proliferation and enhanced osteogenic differentiation in osteoblast cells mediated by integrin α2β1. Moreover, the silencing of α2 or β1 subunits resulted in an increment of cell proliferation and a reduction in cell differentiation comparable to wild-type cells on smoother Ti surfaces [55]. Our results indicate that GBLAST surfaces could enhance the osteoblastic differentiation of hAMSCs. In this context, we examined the differences in α2, α5, αv, β1, β3, and β5 integrin subunit gene expression in hAMSCs between GBLAST and MUCH surfaces. We found an increased expression of α2 and β1 hAMSCs on the GBLAST surface. These results suggest that the Ti surface treated by grit blasting modulates the osteogenic differentiation of hAMSCs via α2/β1 integrin interaction. Further studies need to be performed to reveal the mechanism of osteogenic differentiation of hAMSCs on the GBLAST surface.

Human amniotic stem cells are well-recognized for their advantages, particularly their immunomodulatory capacity and immunoprivileged status, over other types of stem cells for applications in cell therapy [56]. In this regard, hAMSCs have been used in various studies, demonstrating their immunomodulatory and bone regenerative potential [57]. Our results demonstrated the osteogenic differentiation capability of hAMSCSs when cultured on a titanium GBLAST surface. This new finding could be employed in a novel therapeutic strategy to improve the osseointegration of dental implants. In vivo studies will be needed to elucidate the effect of hAMSCs on the local microenvironment of tissue regeneration and osseointegration of Ti GBLAST implants to be used as a potential combined therapy in the implantology field.

## 4. Materials and Methods

### 4.1. Sample Preparation

**Figure 8:**
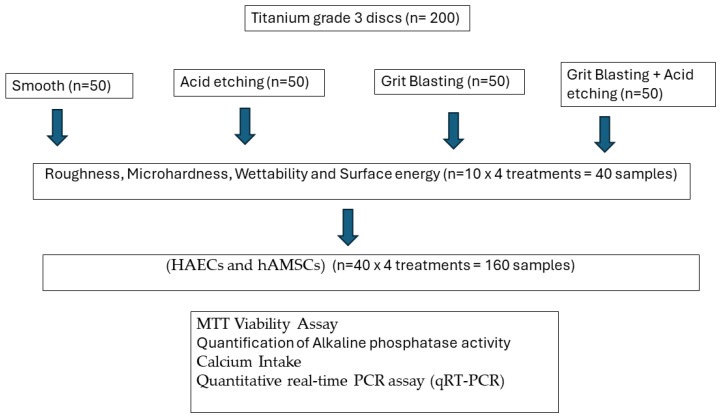
Flowchart of the experiments realized.

Figure 8 shows the flowchart of the tests realized and the number of samples.

Two-hundred titanium discs (5 mm in diameter and 2 mm in width) with different surface treatments were provided by Galimplant^®^ (Sarria, Lugo, Spain). The commercially pure titanium was grade 3 (Ti: 99.5%, O: 0.3%, Fe: 0.1%, C: 0.05%, N: 0.05%). The surfaces were prepared as follows:

- Machined (MACH). The discs show the mechanical abrasion typical of the machining of dental implants without subsequent surface treatment (*n* = 50).
- Grit Blasted (GBLAST). The roughness was obtained by spraying aluminum oxide (Al_2_O_3_) abrasive particles on the titanium surface at a pressure of 2.5 bars and a gun-sample separation of 100 mm (*n* = 50).
- Acid Etching (AE). Acid etching was performed with a mixture of 1:1 concentrated HCl and HNO_3_ acids for 45 s (*n* = 50).
- Grit Blasted and Acid Etching (GBLAST + AE). The discs were blasted with alumina particles of 250 to 450 μm at a pressure of 2.5 bar and at a gun-surface distance of 100 mm. They were then washed with distilled water and treated by immersion in a 1:1 acid mixture of concentrated HCl and HNO_3_ for 45 s (*n* = 50).

These types of topography were developed because they are the most commonly used in titanium hard tissue substitute implants. The machined titanium was the one used as the control. For some applications, especially orthopedic ones, slightly rough titanium obtained by acid etching is used. In this case, there is a double objective to give the titanium a little roughness to facilitate the osteoblastic activity and the generation of a passivation layer to increase the resistance to corrosion and decrease the release of titanium ions to the physiological environment.

The titanium treated by grit blasting with alumina particles generates a higher roughness and also causes a compressive residual stress state on the titanium surface that improves fatigue resistance. This type of treatment is very common in dental implants since a good osseointegration is necessary to cause a biological fixation of the dental implant. It has also been determined that the residual stresses improve the hydrophilicity of the surface, thus improving the protein adsorption activity on the surface, which will facilitate the biological activity on the surface of the implant [5,58,59].

Finally, the combination of grit blasting and acid etching generates a surface suitable for bone tissue growth with good long-term mechanical behavior and also presents a passivation layer that improves its compatibility with chemical degradation.

For the surface characterization studies (roughness and machinability), the samples were cleaned by incubating in methyl alcohol for 15 min in ultrasound and in acetone for 5 min. The materials were dried with hot air flow.

Microhardness tests were performed by Vickers hardness with a high-precision Matzsuzawa microhardness tester (Tokyo, Japan), applying a 1 kg load for 15 s. The indenter was a diamond pyramid.

### 4.2. Characterization of Titanium Disc Surfaces

The SEM (Jeol 6400, Jeol, Tokyo, Japan) was used to observe the topography, and the evaluation of surface roughness was performed by means of Confocal Laser Scanning Microscopy, obtaining the different profiles of the different surfaces (CLSM; OLS Olympus Lext 3000, Shinjuku, Japan). First, the equipment was verified with the use of a reference sample (Mitutoyo SR 15, Elgoibar, Spain “Precision Reference Specimen”: Sa = 0.10 μm). A total of 3 measurements in 3 samples of each surface were calculated [60]. The surfaces observed by Scanning Electron Microscopy are shown in Figure 1.

The contact angle analysis was performed with ultrapure distilled water (Millipore Milli-Q, Merck Millipore Corporation, Darmstadt, Germany) and formamide (Contact Angle System OCA15plus—Dataphysics, Filderstadt, Germany), and the corresponding data were analyzed with SCA20 (Dataphysics, Filderstadt, Germany). Contact angle measurements were made with the sessile drop method. Drops were generated with a micrometric syringe and were deposited over discs. A total of 3 μL of distilled water and 1 μL of formamide were deposited on each sample at 200 μL/min. Wettability is studied by measuring the contact angle follows Young’s equation and describes the balance at the three-phase line between solid (s), liquid (l) and vapor (v):

γsv = γsl + γlvcosΘ_Y_

The interfacial tensions, γsv, γsl and γlv, form the equilibrium contact angle of wetting, referred to as Young’s contact angle Θ_Y_. Young’s equation assumes that the surface is chemically homogenous and topographically smooth. The relationship between roughness and wettability was corrected by Wenzel, who stated that adding surface roughness will enhance the wettability caused by the chemistry of the surface. Wenzel’s statement can be described by:

cosΘm = rcosΘ_Y_

where Θm is the measured contact angle, Θ_Y_ is Young’s contact angle, and r is the roughness ratio. The roughness ratio (r) is defined as the ratio between the actual/real and geometric/nominal solid surface area (r > 1 for a rough surface). The Wenzel equation is an approximation that becomes more accurate as the drop becomes larger compared to the scale of the roughness. Our experimental values were modified using the Wenzel equation [61].

Finally, surface energy was determined by applying the Owens, Wendt, Rabel and Kaelble (OWRK) equation to the wettability values obtained with the distilled water and formamide [8,9]. This energy is calculated based on the two surface tensions: a disperse component and a polar component of the surface free energy.

### 4.3. Isolation and Culture of Human Amniotic Epithelial and Mesenchymal Stem Cells

Human placentas at term were obtained following written informed consent from healthy mothers after normal cesarean deliveries. All procedures used in this study were considered by the ethics committees of the Universitat Internacional Catalunya, and patient consent was signed for the research. All tested methods were conducted in accordance with relevant guidelines and regulations.

The amnion was mechanically separated from the chorion and washed with sterile physiological saline to remove blood and residual connective tissue. The tissue was cut into approximately 6 fragments of 8 cm x 8 cm and sterilized in a laminar flow through successive washing in sterile Phosphate Buffered Saline (PBS) containing penicillin/streptomycin 50 U/mL, amphotericin 50 U/mL, and cephalexin 50 U/mL. Subsequently, the amnion was digested with 0.25% trypsin-EDTA at 37 °C for 20 min. Amniotic epithelial cells (hAECs) were specifically released by this digestion. The remaining cells were centrifuged for 10 min at 1300 rpm. The cell pellet was resuspended and filtered through a sterile 100 μm filter (BD Falcon) to eliminate tissue aggregates. Then, the cell solution was centrifuged for 10 min at 1300 rpm.

To isolate human amniotic mesenchymal stromal cells (hAMSCs), following the separation of hAECs, membrane fragments were digested with 0.75 mg/mL of collagenase II for 90 min at 37 °C with vigorous agitation. The cell suspension was centrifuged for 10 min at 1500 rpm and then filtered. Finally, hAECs and hAMSCs were cultured separately for 24 h in fresh IMDM containing 10% FBS. At the end of each procedure, the total number of isolated cells was determined,

Isolated cells were cultured in IMDM medium containing 10% FBS 4 mM L-glutamine, 1 mM sodium pyruvate, 50 U/mL penicillin, 50 μg/mL streptomycin, and 1 mM non-essential amino acids (Invitrogen, Waltham, MA, USA) at 37 °C in a 5% CO_2_ atmosphere. Medium was changed twice per week.

### 4.4. MTT Viability Assay

HAECs and hAMSCs were seeded in 24-well plates with different titanium discs (2 × 10^5^ cells per well). After the different time incubation, the cells were washed with PBS, and 80 μL of MTT (3-(4,5-dimethylthiazol-2-yl)-2,5-diphenyltetrazolium bromide) (final concentration 1 mg/mL) in 400 μL of DMEM-F12 medium containing 10% FBS was added. The cells were incubated for 30 min at 37 °C. In metabolically active cells, the mitochondrial enzyme succinate dehydrogenase converts MTT into formazan, a blue compound. To quantify the blue color generated, the medium was removed, and the formazan was dissolved in 200 μL of 100% ethanol, followed by incubation at room temperature for 5 min. The absorbance at 570 nm was measured using a DR-200Bs Microplate Reader spectrophotometer (Diatek, South Dumdum, India).

### 4.5. Quantification of Alkaline Phosphatase Activity

Alkaline phosphatase (ALP) activity in hAEC and hAMSC supernatants was quantified with Sensolyte pNPP alkaline phosphatase colorimetric assay (Anaspec, Fremont, CA, USA). The ALP activity was measured at 405 nm in a conventional ELx800 microplate reader (Bio-Tek Instruments, Inc., Winooski, VT, USA).

### 4.6. Calcium Intake

hAMSC and hAEC supernatants were collected (1 mL) and stored at −80 °C. Calcium ion (Ca^2+^) concentrations were determined using the Calcium Assay Kit (Abcam, ab102505) in accordance with the manufacturer’s protocol. In brief, supernatants were centrifuged at top speed for 5 min at 4 °C to eliminate any insoluble material. The resulting supernatants were then transferred to clean tubes. To each sample, 90 μL of the chromogenic reagent was added. The chromogenic complex formed between calcium ions and 0-cresolphthalein was quantified by measuring absorbance at 575 nm using a microplate reader. The absorbance values obtained for each standard were plotted against their final calcium concentration to create a standard curve. The calcium concentrations in the samples were determined by extrapolating from the standard curve. Finally, the calcium intake was calculated by subtracting the calcium concentration of each sample from the total control calcium concentration.

### 4.7. Quantitative Real-Time PCR Assay (qRT-PCR)

To investigate gene expression in both untreated and treated hAECs and hAMSCs, total RNA was isolated using TRISURE reagent following the manufacturer’s instructions (Bioline GmbH, Berlin, Germany). The concentration and purity of the isolated RNA were determined spectrophotometrically at wavelengths of 260 nm and 280 nm in a NanoDrop ND-1000 Spectrophotometer (NanoDrop Technologies, Wilmington, DC, USA).

For complementary DNA (cDNA) synthesis, 5 μg of total RNA was subjected to reverse transcription at 50 °C for 1 h, employing the Transcriptor First Strand cDNA Synthesis Kit (Roche). Subsequently, a qRT-PCR reaction was carried out using forward and reverse primers designed specifically for the gene of interest: ALP (F:5’-ACTCCCACTTCATCTGGAACC-3′, R:5′-CCTGTTCAGCTCGTACTGCAT-3′), RUNX2 (F:5′-GTCTCACTGCCTCTCACTTG-3′, R:5′-CACACATCTCCTCCCTTCTG-3′), OSTERIX (F:5′-TGAGGAGGAAGTTCACTATGG-3′, R:5′-TTCTTTGTGCCTGCTTTGC-3′), OPN (F:5′-CAGAATGCTGTGTCCTCTGAA-3′, R:5′-GTCAATGGAGTCCTGGCTGT-3′), α2 INTEGRIN (F:5′-CCTACAATGTTGGTCTCCCAGA-3′, R:5′-AGTAACCAGTTGCCTTTTGGATT-3′), α5 INTEGRIN (F:5′-GGCTTCAACTTAGACGCGGAG-3′, R:5′-TGGCTGGTATTAGCCTTGGGT-3′), αv INTEGRIN (F:5′-ATCTGTGAGGTCGAAACAGGA-3′, R:5′-TGGAGCATACTCAACAGTCTTTG-3′), β1 INTEGRIN (F:5′-CCTACTTCTGCACGATGTGATG-3′, R:5′-CCTTTGCTACGGTTGGTTACATT-3′), β3 INTEGRIN (F:5′-TCCTCATCACCATCCACGA-3′, R:5′-TTATCAGCCTGTGCCACGA-3′), β5 INTEGRIN (F:5′-TCTCGGTGTGATCTGAGGG-3′, R:5′-TGGCGAACCTGTAGCTGGA-3′), CYCLOPHILIN (F:5′-CTTCCCCGATACTTCA-3′, R:5′-TCTTGGTGCTACCTC-3′), and GAPDH (F:5′-TCCCTGAGCTGAACGGGAAG-3′, R:5′-GGAGGAGTGGGTGTCGCTGT-3′). Quantitative RT-PCR (qRT-PCR) analysis was conducted using a SYBR Premix Ex Taq kit (Takara BIO Inc., Shiga, Japan), and the PCR reactions were executed on an Applied Biosystems 7500 RT-PCR System (Applied Biosystems, Foster City, CA, USA). Each standard reaction consisted of 1 μM of both forward and reverse primers, 9 µL of SYBR Green master mix 2X, 1 μL of cDNA and water, resulting in a final reaction volume of 15 μL. The thermal cycling process consisted of 95 °C for 10 min, 95 °C for 30 s, 60 °C for 10 min for 40 amplification cycles, and annealing/extension at 60 °C for 1 min. The Opticon Monitor 3 Program was employed to determine the threshold cycle (CT) for each well. Relative quantification was determined using the 2^−∆∆CT^ technique. In the case of treated samples, the 2^−∆∆CT^ value indicated the fold change in gene expression, normalized against housekeeping genes (CYCLOPHILIN and GAPDH).

### 4.8. Statistical Analysis

Results are presented as the average ± the standard error of the average (SEM) for a minimum of four independent experiments. Statistical comparisons among the data were performed using two-way analysis of variance (ANOVA). Statistical significance was defined as having a *p* ≤ 0.05.

## 5. Conclusions

Dental implants treated by grit blasting show a higher roughness as well as a higher contact angle and lower surface energy when compared to chemically treated or machined surfaces. The projection of alumina on the titanium surface increases the surface hardness due to the compressive loads on the surface. We have shown that amnion mesenchymal stem cells have higher cell viability and a higher degree of osteogenic differentiation at 1, 7 and 14 days for surfaces treated with grit blasting compared to acid-etched and machined surfaces. HAECs does not seem to differentiate into osteoblastic cells given that we have not observed osteoblastic gene expression or mineralization signs. Therefore, hAMSCs but not hAECs would be suitable for the osseointegration process. Further in vitro and in vivo studies are required to assess the impact of hAMSCs on implant osseointegration. Nevertheless, this novel study offers new insights into the behavior of amniotic stem cells on different Ti surfaces. These findings will enable further research into new therapeutic strategies and applications in oral implantology.

## Figures and Tables

**Table 1:** Description of surface roughness (Sa), roughness ratio (r), and contact angle are shown in mean ± standard deviation. Asterisks mean statistical difference significance with *p* < 0.001. The values of the contact angle were modified following the Wenzel equation.

Surfaces	Roughness		Contact Angle (◦)	
	(Sa) (µm)	r	H_2_O	Formamide
MACH	0.22 ± 0.09 *	1.09 ± 0.09 *	53.4 ± 6.1 *	31.6 ± 4.3 *
GBLAST	2.06 ± 0.20 **	1.66 ± 0.32 **	89.5 ± 9.9 **	63.2 ± 10.3 **
AE	1.28 ± 0.17 *	1.27 ± 0.25 *	59.4 ± 2.2 *	36.6 ± 6.2 *
GBLAST + AE	2.22 ± 0.19 **	1.76 ± 0.27 **	92.3 ± 4.9 **	70.2 ± 12.3 **

**Table 2:** Water contact angle, surface free energy and its components for the different Ti surfaces. Values are mean ± standard error of the mean. Statistical differences vs. smooth surfaces for each column are indicated by single and double asterisk symbols (*p* < 0.05).

Surface	Surface Free Energy (mJ/m^2^)		
	Total Surface Free Energy	Dispersive Component	Polar Component
MACH	49.6 ± 3.3 *	30.20 ± 4.32 *	19.40 ± 2.88 *
GBLAST	38.8 ± 4.0 **	14.20 ± 2.21 *	24.60 ± 1.50 **
AE	46.5 ± 3.5 *	30.99 ± 0.85 *	15.51 ± 2.90 **
GBLAST + AE	39.3 ± 2.7 **	12.10 ± 2.34 *	27.20 ± 5.15 *

## Data Availability

The authors can provide details of the research upon request by letter and commenting on their needs.

## References

[1] Albrektsson T., Wennerberg A. (2019). On osseointegration in relation to implant surfaces. Clin. Implant. Dent. Relat. Res..

[2] Blatt S., Pabst A.M., Schiegnitz E., Hosang M., Ziebart T., Walter C., Al-Nawas B., Klein M.O. (2018). Early cell response of osteogenic cells on differently modified implant surfaces: Sequences of cell proliferation, adherence and differentiation. J. Cranio-Maxillofac. Surg..

[3] Albrektsson T., Branemark P.I., Hansson H.A., Lindstrom J. (1981). Osseointegrated titanium implants. Requirements for ensuring a long-lasting, direct bone-to-implant anchorage in man. Acta Orthop. Scand..

[4] Pellegrini G., Francetti L., Barbaro B., Del Fabbro M. (2018). Novel surfaces and osseointegration in implant dentistry. J. Investig. Clin. Dent..

[5] Velasco-Ortega E., Alfonso-Rodriguez C.A., Monsalve-Guil L., Espana-Lopez A., Jimenez-Guerra A., Garzon I., Alaminos M., Gil F.J. (2016). Relevant aspects in the surface properties in titanium dental implants for the cellular viability. Mater. Sci. Eng. C Mater. Biol. Appl..

[6] Pegueroles M., Aparicio C., Bosio M., Engel E., Gil F.J., Planell J.A., Altankov G. (2010). Spatial organization of osteoblast fibronectin matrix on titanium surfaces: Effects of roughness, chemical heterogeneity and surface energy. Acta Biomater..

[7] Lange R., Luthen F., Beck U., Rychly J., Baumann A., Nebe B. (2002). Cell-extracellular matrix interaction and physico-chemical characteristics of titanium surfaces depend on the roughness of the material. Biomol. Eng..

[8] Annarelli C.C., Fornazero J., Cohen R., Bert J., Besse J. (1999). Colloidal Protein Solutions as a New Standard Sensor for Adhesive Wettability Measurements. J. Colloid. Interface Sci..

[9] Lukaszewska-Kuska M., Wirstlein P., Majchrowski R., Dorocka-Bobkowska B. (2018). Osteoblastic cell behaviour on modified titanium surfaces. Micron.

[10] Aparicio C., Gil F.J., Fonseca C., Barbosa M., Planell J.A. (2003). Corrosion behaviour of commercially pure titanium shot blasted with different materials and sizes of shot particles for dental implant applications. Biomaterials.

[11] Ferraris S., Truffa Giachet F., Miola M., Bertone E., Varesano A., Vineis C. (2017). Nanogrooves and keratin nanofibers on titanium surfaces aimed at driving gingival fibroblasts alignment and proliferation without increasing bacterial adhesion. Mater. Sci. Eng. C Mater. Biol. Appl..

[12] Zhang J., Liu J., Wang C., Chen F., Wang X., Lin K. (2020). A comparative study of the osteogenic performance between the hierarchical micro/submicro-textured 3D-printed Ti6Al4V surface and the SLA surface. Bioact. Mater..

[13] Velasco E., Monsalve-Guil L., Jimenez A., Ortiz I., Moreno-Munoz J., Nunez-Marquez E., Pegueroles M., Pérez R.A., Gil F.J. (2016). Importance of the Roughness and Residual Stresses of Dental Implants on Fatigue and Osseointegration Behavior. In Vivo Study in Rabbits. J. Oral. Implantol..

[14] Gil J., Perez R., Herrero-Climent M., Rizo-Gorrita M., Torres-Lagares D., Gutierrez J.L. (2021). Benefits of Residual Aluminum Oxide for Sand Blasting Titanium Dental Implants: Osseointegration and Bactericidal Effects. Materials.

[15] Gil F.J., Herrero-Climent M., Lazaro P., Rios J.V. (2014). Implant-abutment connections: Influence of the design on the microgap and their fatigue and fracture behavior of dental implants. J. Mater. Sci. Mater. Med..

[16] Gil F.J., Planell J.A., Padros A., Aparicio C. (2007). The effect of shot blasting and heat treatment on the fatigue behavior of titanium for dental implant applications. Dent. Mater..

[17] Cervino G., Fiorillo L., Iannello G., Santonocito D., Risitano G., Cicciu M. (2019). Sandblasted and Acid Etched Titanium Dental Implant Surfaces Systematic Review and Confocal Microscopy Evaluation. Materials.

[18] Velasco-Ortega E., Ortiz-Garcia I., Jimenez-Guerra A., Nunez-Marquez E., Moreno-Munoz J., Rondon-Romero J.L., Cabanillas-Balsera D., Gil J., Muñoz-Guzón F., Monsalve-Guil L. (2021). Osseointegration of Sandblasted and Acid-Etched Implant Surfaces. A Histological and Histomorphometric Study in the Rabbit. Int. J. Mol. Sci..

[19] Zareidoost A., Yousefpour M., Ghaseme B., Amanzadeh A. (2012). The relationship of surface roughness and cell response of chemical surface modification of titanium. J. Mater. Sci. Mater. Med..

[20] Godoy-Gallardo M., Guillem-Marti J., Sevilla P., Manero J.M., Gil F.J., Rodriguez D. (2016). Anhydride-functional silane immobilized onto titanium surfaces induces osteoblast cell differentiation and reduces bacterial adhesion and biofilm formation. Mater. Sci. Eng. C Mater. Biol. Appl..

[21] Guillem-Marti J., Cinca N., Punset M., Cano I.G., Gil F.J., Guilemany J.M., Dosta S. (2019). Porous titanium-hydroxyapatite composite coating obtained on titanium by cold gas spray with high bond strength for biomedical applications. Colloids Surf. B Biointerfaces.

[22] Pereira R., Maia P., Rios-Santos J.V., Herrero-Climent M., Rios-Carrasco B., Aparicio C., Gil J. (2024). Influence of Titanium Surface Residual Stresses on Osteoblastic Response and Bacteria Colonization. Materials.

[23] Dutta S.R., Passi D., Singh P., Atri M., Mohan S., Sharma A. (2020). Risks and complications associated with dental implant failure: Critical update. Natl. J. Maxillofac. Surg..

[24] Bardis D., Agop-Forna D., Pelekanos S., Chele N., Dascalu C., Torok R., Török B., Cristea I., Bardi P.M., Forna N. (2023). Assessment of Various Risk Factors for Biological and Mechanical/Technical Complications in Fixed Implant Prosthetic Therapy: A Retrospective Study. Diagnostics.

[25] Perrotti V., Palmieri A., Pellati A., Degidi M., Ricci L., Piattelli A., Carinci F. (2013). Effect of titanium surface topographies on human bone marrow stem cells differentiation in vitro. Odontology.

[26] Barone A., Toti P., Bertossi D., Marconcini S., De Santis D., Nocini P.F., Iurlaro A., Alfonsi F., Covani U. (2016). Gene Expression of Human Mesenchymal Stem Cells Cultured on Titanium Dental Implant Surfaces. J. Craniofac. Surg..

[27] Choi H., Park K.H., Jung N., Shim J.S., Moon H.S., Kim H.J., Ku S.Y., Park Y.B. (2021). In Vivo Study for Clinical Application of Dental Stem Cell Therapy Incorporated with Dental Titanium Implants. Materials.

[28] Li H., Huang J., Wang Y., Chen Z., Li X., Wei Q., Liu X., Wang Z., Wen B., Zhao Y. (2022). Nanoscale Modification of Titanium Implants Improves Behaviors of Bone Mesenchymal Stem Cells and Osteogenesis In Vivo. Oxidative Med. Cell. Longev..

[29] Long E.G., Buluk M., Gallagher M.B., Schneider J.M., Brown J.L. (2019). Human mesenchymal stem cell morphology, migration, and differentiation on micro and nano-textured titanium. Bioact. Mater..

[30] Tseng K.F., Shiu S.T., Hung C.Y., Chan Y.H., Chee T.J., Huang P.C., Lai P.C., Feng S.W. (2024). Osseointegration Potential Assessment of Bone Graft Materials Loaded with Mesenchymal Stem Cells in Peri-Implant Bone Defects. Int. J. Mol. Sci..

[31] Saito S., Lin Y.C., Murayama Y., Hashimoto K., Yokoyama K.K. (2012). Human amnion-derived cells as a reliable source of stem cells. Curr. Mol. Med..

[32] Magatti M., Vertua E., Cargnoni A., Silini A., Parolini O. (2018). The Immunomodulatory Properties of Amniotic Cells: The Two Sides of the Coin. Cell Transplant..

[33] Luan F., Ma K., Mao J., Yang F., Zhang M., Luan H. (2018). Differentiation of human amniotic epithelial cells into osteoblasts is induced by mechanical stretch via the Wnt/β-catenin signalling pathway. Mol. Med. Rep..

[34] Wang Q., Wu W., Han X., Zheng A., Lei S., Wu J., Chen H., He C., Luo F., Liu X. (2014). Osteogenic differentiation of amniotic epithelial cells: Synergism of pulsed electromagnetic field and biochemical stimuli. BMC Musculoskelet. Disord..

[35] Hendrijantini N., Hartono P. (2019). Phenotype Characteristics and Osteogenic Differentiation Potential of Human Mesenchymal Stem Cells Derived from Amnion Membrane (HAMSCs) and Umbilical Cord (HUC-MSCs). Acta Inform. Medica.

[36] Xing Y., Zhang M.S., Xiao J.H., Liu R.M. (2022). Galangin induces the osteogenic differentiation of human amniotic mesenchymal stem cells via the JAK2/STAT3 signaling pathway. Eur. J. Pharmacol..

[37] Chen P., Lu M., Wang T., Dian D., Zhong Y., Aleahmad M. (2021). Human amniotic membrane as a delivery vehicle for stem cell-based therapies. Life Sci..

[38] Balshi T.J., Wolfinger G.J., Balshi S.F., Bidra A.S. (2019). A 30-Year Follow-Up of a Patient with Mandibular Complete-Arch Fixed Implant-Supported Prosthesis on 4 Implants: A Clinical Report. J. Prosthodont..

[39] Zhao G., Schwartz Z., Wieland M., Rupp F., Geis-Gerstorfer J., Cochran D.L., Cochran B.D. (2005). Boyan. High surface energy enhances cell response to titanium substrate microstructure. J. Biomed. Mater. Res. A.

[40] Velasco-Ortega E., Fos-Parra I., Cabanillas-Balsera D., Gil J., Ortiz-Garcia I., Giner M., Jiménez-Guerra Á. (2023). Osteoblastic Cell Behavior and Gene Expression Related to Bone Metabolism on Different Titanium Surfaces. Int. J. Mol. Sci..

[41] Nicolas-Silvente A.I., Velasco-Ortega E., Ortiz-Garcia I., Monsalve-Guil L., Gil J., Jimenez-Guerra A. (2020). Influence of the Titanium Implant Surface Treatment on the Surface Roughness and Chemical Composition. Materials.

[42] Palmquist A., Omar O.M., Esposito M., Lausmaa J., Thomsen P. (2010). Titanium oral implants: Surface characteristics, interface biology and clinical outcome. J. R. Soc. Interface..

[43] Murphy M., Walczak M.S., Thomas A.G., Silikas N., Berner S., Lindsay R. (2017). Toward optimizing dental implant performance: Surface characterization of Ti and TiZr implant materials. Dent. Mater..

[44] Yin L., Chang Y., You Y., Liu C., Li J., Lai H.C. (2019). Biological responses of human bone mesenchymal stem cells to Ti and TiZr implant materials. Clin. Implant. Dent. Relat. Res..

[45] Park J.W., Tsutsumi Y., Park E.K. (2022). Osteogenic Differentiation of Human Mesenchymal Stem Cells Modulated by Surface Manganese Chemistry in SLA Titanium Implants. Biomed. Res. Int..

[46] Wang W., Wang Z., Fu Y., Dunne N., Liang C., Luo X., Liu K., Li X., Pang X., Lu K. (2020). Improved osteogenic differentiation of human amniotic mesenchymal stem cells on gradient nanostructured Ti surface. J. Biomed. Mater. Res. A.

[47] Sola-Ruiz M.F., Perez-Martinez C., Labaig-Rueda C., Carda C., Martin De Llano J.J. (2017). Behavior of Human Osteoblast Cells Cultured on Titanium Discs in Relation to Surface Roughness and Presence of Melatonin. Int. J. Mol. Sci..

[48] Vansana P., Kakura K., Taniguchi Y., Egashira K., Matsuzaki E., Tsutsumi T., Kido H. (2022). The effect of AMP kinase activation on differentiation and maturation of osteoblast cultured on titanium plate. J. Dent. Sci..

[49] Si J., Dai J., Zhang J., Liu S., Gu J., Shi J., Shen S.G., Guo L. (2015). Comparative investigation of human amniotic epithelial cells and mesenchymal stem cells for application in bone tissue engineering. Stem Cells Int..

[50] Park B.H., Jeong E.S., Lee S., Jang J.H. (2021). Bio-functionalization and in-vitro evaluation of titanium surface with recombinant fibronectin and elastin fragment in human mesenchymal stem cell. PLoS ONE.

[51] Yin L., Zhou Z.X., Shen M., Chen N., Jiang F., Wang S.L. (2019). The Human Amniotic Mesenchymal Stem Cells (hAMSCs) Improve the Implant Osseointegration and Bone Regeneration in Maxillary Sinus Floor Elevation in Rabbits. Stem Cells Int..

[52] Yang J., Wang J., Yuan T., Zhu X.D., Xiang Z., Fan Y.J., Zhang X.D. (2013). The enhanced effect of surface microstructured porous titanium on adhesion and osteoblastic differentiation of mesenchymal stem cells. J. Mater. Sci. Mater. Med..

[53] Humphries M.J., Travis M.A., Clark K., Mould A.P. (2004). Mechanisms of integration of cells and extracellular matrices by integrins. Biochem. Soc. Trans..

[54] Olivares-Navarrete R., Hyzy S.L., Hutton D.L., Erdman C.P., Wieland M., Boyan B.D., Schwartz Z. (2010). Direct and indirect effects of microstructured titanium substrates on the induction of mesenchymal stem cell differentiation towards the osteoblast lineage. Biomaterials.

[55] Lai M., Hermann C.D., Cheng A., Olivares-Navarrete R., Gittens R.A., Bird M.M., Walker M., Cai Y., Cai K., Sandhage K.H. (2015). Role of α2β1 integrins in mediating cell shape on microtextured titanium surfaces. J. Biomed. Mater. Res. A..

[56] Liu Q.W., Huang Q.M., Wu H.Y., Zuo G.S., Gu H.C., Deng K.Y., Xin H.B. (2021). Characteristics and Therapeutic Potential of Human Amnion-Derived Stem Cells. Int. J. Mol. Sci..

[57] Li J., Zhou Z., Wen J., Jiang F., Xia Y. (2020). Human Amniotic Mesenchymal Stem Cells Promote Endogenous Bone Regeneration. Front. Endocrinol..

[58] Manero J.M., Gil F.J., Padros E., Planell J.A. (2003). Applications of environmental scanning electron microscopy (ESEM) in biomaterials field. Microsc. Res. Tech..

[59] Gil F.J., Solano E., Pena J., Engel E., Mendoza A., Planell J.A. (2004). Microstructural, mechanical and citotoxicity evaluation of different NiTi and NiTiCu shape memory alloys. J. Mater. Sci. Mater. Med..

[60] (2001). Surface Roughness Standards.

[61] Wenzel R.W. (1936). Resistance of solid surfaces to wetting by water. Ind. Eng. Chem..

